# Maternal and late mortality trends, emphasizing the H1N1 and COVID-19 pandemics, in the state of Rio de Janeiro, Brazil, from 2009 to 2021

**DOI:** 10.1590/1980-549720250037

**Published:** 2025-07-21

**Authors:** Ana Lucia de Melo Bellizzi, Angela Maria Cascão, Alexandre dos Santos Brito, Sandra Costa Fonseca, Pauline Lorena Kale

**Affiliations:** I Universidade Federal do Rio de Janeiro, Institute for Collective Health Studies, Graduate Program - Rio de Janeiro (RJ), Brazil.; II Secretaria Estadual de Saúde do Rio de Janeiro, Undersecretariat for Health Surveillance - Rio de Janeiro (RJ), Brazil.; III Universidade Federal do Rio de Janeiro, Institute for Collective Health Studies - Rio de Janeiro (RJ), Brazil.; IV Universidade Federal Fluminense, Institute of Collective Health - Niterói (RJ), Brazil.

**Keywords:** Time series studies, Maternal mortality, Influenza A virus, H1N1 subtype, COVID-19

## Abstract

**Objective:**

Trends in maternal mortality (MMR) and late maternal mortality ratios (LMMR) were estimated, in periods with and without H1N1 and COVID-19 pandemics, in Rio de Janeiro, Brazil, from 2009 to 2021.

**Methods:**

Ecological study of temporal trends. Data was obtained from the Mortality and Live Birth Information Systems. The annual MMR and LMMR per 100,000 live births (LB) were calculated and the trends were estimated using the joinpoint regression model.

**Results:**

In 2009, the MMR was 103.1, reaching 152.4/100,000 LB in 2021, with an annual reduction of 3.3% (95% confidence interval - 95%CI -5.5; -1.7) until 2019 and an increase of 51.2% (95%CI 23.5; 64.5) in 2020/21. Excluding the years of the COVID-19 pandemic, it was observed that an annual decline of 3.3% and, with the concomitant exclusion of the years of the H1N1 pandemic, stability. The LMMR were 8.3 (2009) and 22.2 (2021) per 100,000 LB, with an annual growth of 28.2% (95%CI 11.8; 47.8) until 2011, remaining stationary from 2011 to 2015, followed by an increase of 11.7% until 2021; with the exclusion of the final biennium, the trend is upward (3.8%) and also with the exclusion of the initial biennium, the trend became downward (7%) until 2014 and upward (8.2%) from then on.

**Conclusion:**

There was a change in trend with the separate or joint incorporation of pandemic biennia: without pandemics, maternal mortality would be stationary, despite actions to prevent maternal deaths, and late maternal mortality, would be descending until 2014 and then ascending, crediting itself in part, to improving death investigation.

## INTRODUCTION

Globally, one woman dies every two minutes due to complications related to pregnancy or childbirth^1^, corresponding to a maternal mortality ratio (MMR - defined as the risk of maternal death during pregnancy or within 42 days postpartum) of 223 per 100,000 live births (LB) in 2020^2^. In the same year, Brazil reported an MMR of 72 per 100,000 LB, approximately 16 times higher than Germany and eight times higher than Cuba, highlighting a significant potential for reduction^3^. Between 2000 and 2020, the average annual decrease in the global MMR was 2.1%, whereas in Latin America and the Caribbean, the trend remained stagnant^3^.

The H1N1 influenza pandemics in 2009 and 2010, as well as the COVID-19 (SARS-CoV-2) pandemic approximately a decade later (beginning in Brazil in 2020), affected maternal mortality rates^4^ ^,^ ^5^ ^,^ ^6^, although they do not fully account for the observed trends^3^. Due to anatomical and physiological changes in the cardiovascular, respiratory, immune, and coagulation systems during pregnancy, women are more susceptible to viral pneumonias, particularly during pandemics, which exacerbate preexisting challenges within healthcare systems^6^ ^,^ ^7^.

Sustained transmission of the influenza A (H1N1) virus was officially declared nationwide on July 19^th^, 2009. That year, nearly 60,000 cases and 2,146 deaths were reported in the country. In 2010, following the introduction of vaccination, the number of deaths declined to approximately one hundred^8^ ^,^ ^9^. More than 10% of total deaths caused by the H1N1 pandemic occurred among pregnant women, most of whom had no underlying health conditions^10^. In the first quarter of 2010, the monovalent anti-H1N1 vaccine was distributed across the country^9^. A decade later, by June 2020, Brazil had recorded 124 deaths among women in the pregnancy-puerperal cycle due to COVID-19, representing 12.7% of deaths among pregnant women hospitalized for severe acute respiratory syndrome (SARS)^11^. COVID-19 was associated with 18% and 43% of national maternal deaths in 2020 and 2021, respectively^12^. Complete COVID-19 immunization during pregnancy was shown to be highly protective against severe maternal morbidity and mortality in a cohort of pregnant women treated at a public general hospital in the state of Rio de Janeiro^13^. In both viral pandemics, women in the pregnancy-puerperal cycle were considered a high-risk group for severe maternal morbidity and mortality^11^ ^,^ ^12^ ^,^ ^13^. According to Vega et al.^14^, pandemic situations should not be considered in the analysis of historical maternal mortality trends, as they are exceptional events, comparable to major catastrophes, that function as external factors, independent of public health management, affecting populations unprepared to respond effectively^14^.

With advancements in life support technology, the 42-day postpartum limit traditionally used to define maternal mortality has become increasingly inadequate^15^. In the United States, the inclusion of information on pregnancy within the year preceding death on death certificates (DC) has contributed to improved detection of late maternal mortality (from 43 days to one year after delivery)^16^.

In Brazil, beyond the incorporation of pregnancy-related information into DC, the epidemiological investigation of deaths among women of childbearing age and maternal deaths, implemented since 2008, has played a key role in reducing the underreporting of maternal and late maternal deaths^17^ ^,^ ^18^. Between 2010 and 2019, the country experienced an upward trend in late maternal mortality, increasing from 2.2 to 5.6/100,000 LB, with an annual growth rate of 9.8% and notable regional disparities^19^. In the city of São Paulo (SP) and the state of Paraná, late maternal deaths accounted for 13.4 and 12.1% of total maternal deaths, respectively^17^. These deaths are largely preventable through timely and effective treatment, underscoring the importance of extending postpartum monitoring, particularly for women at higher risk^17^ ^,^ ^18^ ^,^ ^19^. Failure to address late maternal deaths contributes to fragmented care and missed opportunities for prevention^20^.

This study aimed to analyze trends in MMR and late maternal mortality (LMMR) during periods with and without the influenza A (H1N1) and COVID-19 pandemics in the state of Rio de Janeiro, from 2009 to 2021.

## METHODS

An ecological study was conducted to analyze the temporal trends of the annual MMR and LMMR in the state of Rio de Janeiro from 2009 to 2021.

Maternal deaths are defined as those occurring during pregnancy, childbirth, or within 42 days following the end of pregnancy, regardless of the duration or location of the pregnancy, resulting from any cause related to or aggravated by the pregnancy or its management, but not from accidental or incidental causes^21^. The following ICD-10 codes are classified as causes of maternal death: O00.0 to O08.9, O11 to O23.9, O24.4, O26.0 to O92.7, D39.2, E23.0, F53, and M83.0 (direct obstetric causes), O10.0 to O10.9, O24.0 to O24.3, O24.9, O25, O98.0 to O99.8, A34, and B20 to B24 (indirect obstetric causes), and O95 (unspecified causes), all from Chapter XV of the ICD-10. Additionally, codes A34, B20 to B24, D39.2, E23.0, F53, and M83.0 are included when they appear outside Chapter XV but result in death during the pregnancy-puerperal cycle^21^.

Late maternal deaths are defined as deaths from any obstetric cause occurring more than 42 days but less than one year after the end of pregnancy, corresponding to code O96 of the International Statistical Classification of Diseases and Related Health Problems, Tenth Revision (ICD-10)^21^.

Secondary data were obtained from the Mortality Information System (*Sistemas de Informações sobre Mortalidade* - SIM) and the Live Birth Information System (*Sistemas de Informações sobre Nascidos Vivos* - Sinasc) of the Rio de Janeiro State Health Secretariat.

The absolute and relative percentage distributions of maternal and late maternal deaths were described annually. The annual MMR and LMMR were calculated per 100,000 LB (using the ratio of maternal deaths to LB, multiplied by 100,000).

The decision to begin the temporal analysis in 2009 was based on the exclusion of a correction factor, which was only applied to MMR in the preceding period in the state of Rio de Janeiro^4^. The maternal mortality time series were evaluated for the entire period (2009 to 2021), as well as for periods excluding the initial biennium of the influenza A/H1N1 pandemic (2011 to 2021), the final biennium of the COVID-19 pandemic (2009 to 2019), and both pandemic biennia (2011 to 2019).

To estimate the trend, a joinpoint regression model was applied, which detects significant changes in the trend (inflection points) and fits linear segments on a logarithmic scale^22^ ^,^ ^23^ ^,^ ^24^. Model selection (including the number of joinpoints) was based on the weighted Bayesian Information Criterion (W-BIC). The annual percentage change (APC) was calculated to represent the direction and magnitude of the trend (significance level set at 0.05). Analyses were conducted both in the absence of autocorrelation and considering first-order serial autocorrelation. The model that best described the trend structure of the historical series was selected.

This study is part of a research project approved by the Research Ethics Committee of Universidade Federal Fluminense (Certificate of Presentation for Ethical Consideration - CAAE No. 713230230000005243; approval opinion No. 6.592.725, dated December 19, 2023).

### Data availability statement

The entire dataset supporting the results of this study was published in the article itself.

## RESULTS

In 2009, the number of LB in the state of Rio de Janeiro was 217,280, decreasing to 188,989 in 2021, a reduction of 13% (Table 1). It is noteworthy, however, that there was a slight increase of 8.5% in the number of LB up to 2015, followed by a decline of 25.7% thereafter (Table 1).

**Table 1.** Absolute and percentage distribution of maternal and late maternal deaths, per live births, maternal mortality ratio* and late maternal mortality ratio^†^, state of Rio de Janeiro, 2000 to 2021.

Year	No. of live births	No. of maternal deaths (total)	Maternal mortality*			Late maternal mortality^†^		
			No. deaths	% deaths	MMR	No. deaths	% deaths	LMMR
2009	217,280	242	224	92.6	103.1	18	7.4	8.3
2010	215,916	211	189	89.6	87.5	22	10.4	10.2
2011	221,277	197	164	83.2	74.1	33	16.8	14.9
2012	223,422	212	186	87.7	83.3	26	12.3	11.6
2013	224,745	221	193	87.3	85.9	28	12.7	12.5
2014	234,281	202	175	86.6	74.7	27	13.4	11.5
2015	237,591	193	167	86.5	70.3	26	13.5	10.9
2016	219,711	185	156	84.3	71.0	29	15.7	13.2
2017	223,415	205	173	84.4	77.4	32	15.6	14.3
2018	221,045	174	136	78.2	61.5	38	21.8	17.2
2019	208,677	177	149	84.2	71.4	28	15.8	13.4
2020	199,550	229	187	81.7	93.7	42	18.3	21
2021	188,989	330	288	87.3	152.4	42	12.7	22.2

MMR: Maternal mortality ratio per 100,000 live births and late maternal mortality ratio per 100,00 live births.

*Causes of maternal death from Chapter XV and outside of Chapter XV - ICD-10 up to 42 days after childbirth; 10: O96); ^†^Late Maternal Death - from 43 days up to less than one year after childbirth (ICD-10: O96).

Source: Mortality Information System and Live Births System.

There were 242 maternal deaths (including late maternal deaths) at the beginning of the study period and 330 at the end, with the lowest number recorded in 2018 (174 deaths) and the highest in 2021 (Table 1). Most maternal deaths occurred within 42 days postpartum; however, there was a notable increase in the proportion of late maternal deaths (occurring between 43 days and one year postpartum), rising from 7.4% in 2009 to 18.3% in 2020. The highest value of LMMR prior to the COVID-19 pandemic was observed in 2018. The MMR showed the highest values in the initial and final biennia of the study period, ranging from 61.5 (2018) to 152.4 (2021) per 100,000 LB (Table 1).

The estimated trends, joinpoints (changes in trend), and the APC coefficients for the MMR and LMMR, with and without the inclusion of pandemic years, are summarized in Table 2. These estimates were derived considering first-order autocorrelation, as this approach provided a better fit for the trend observed in the historical series.

**Table 2.** Trend of maternal mortality ratio and late maternal mortality ratio per 100,000 live births, Rio de Janeiro state, 2009 to 2021.

Period	Joinpoint	Segments	Mortality ratio		Annual Percentage Variation (95%CI)*	Trend
			Start	End		
Maternal^†^						
2009-2021	2019	2009-2019	103.1	71.4	-3.3 (-5.5; -1.7)	descending
		2019-2021	71.4	152.4	51.2 (23.5; 64.5)	ascending
2011-2021	2019	2011-2019	74.1	71.4	-2.7 (-5.7; 0.5)	descending
		2019-2021	71.4	152.4	49.0 (23.5; 62.6)	ascending
2009-2019	-	-	103.1	71.4	- 3.3 (-5.4; -1.3)	descending
2011-2019	-	-	74.1	71.4	- 2.6 (-5.5; 0.2)	stationary
Late^‡^						
2009-2021	2009	2009-2011	8.3	14.9	28.3 (11.8; 47.8)	ascending
	2011	2011-2015	14.9	10.9	-4.6 (-11.7; 0.4)	stationary
	2015	2015-2021	10.9	22.2	11.7 (8.7; 17.6)	ascending
2011-2021	2015	2011-2015	14.9	10.9	- 4.4 (-17.9; 2.2)	stationary
		2015-2021	10.9	22.2	11.7 (7.9; 20.4)	ascending
2009-2019	-	-	8.3	13.4	3.8 (0.2; 8.1)	ascending
2011-2019	2014	2011-2014	14.9	11.5	- 7.0 (-16.8; -0.9)	descending
		2014-2019	11.5	13.4	8.2 (4.7; 15.4)	ascending

*95%CI: 95% confidence interval; ^†^Causes of maternal death from Chapter XV and outside of Chapter XV - ICD-10 up to 42 days after childbirth; 10: O96); ^‡^Late maternal death - from 43 days up to less than one year after childbirth (CID-10: O96).

Source: Mortality Information System and Live Births System.

During the full period from 2009 to 2021, the MMR exhibited a segmented trend, with an annual decline of 3.3% until 2019 (joinpoint), followed by an annual increase of 51.2% in the final two years (COVID-19 pandemic period - Table 2). When excluding the initial two years, the trend remained downward until 2019 (APC=-2.7%), followed by an upward trend from that point onward (APC=49%). When excluding the final two years, an annual decline of 3.3% was observed, and when both pandemic biennia (initial and final) were excluded, the trend remained stable (Table 2).

For the LMMR, considering the full period, three trend changes were identified: an annual increase of 28.3% until 2011, followed by a stationary period, and, from 2015 onward, an annual increase of 11.7% (Table 2). When excluding the initial two-year period, the trend remained stationary until 2015, followed by an upward trend (APC=11.7%). Excluding the final two years, no joinpoint was observed, and the trend showed a consistent annual increase (APC=3.8%). When both the initial and final biennia were excluded, a decline of 7.0% per year was observed until 2014, followed by an annual increase of 8.2% through 2019 (Table 2).

**Figure 1.**
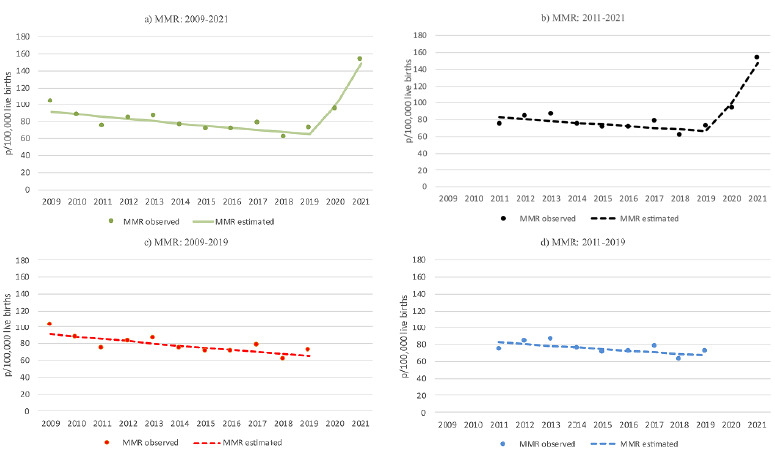
Trend of maternal mortality ratio per 100,000 live births, Rio de Janeiro State, 2009 to 2021.

Figures 1 (MMR) and 2 (LMMR) present the graphical representations of the observed and estimated trends in maternal and late maternal mortality from 2009 to 2021, as well as the simulated trends in the absence of pandemic periods.

MMR: maternal mortality ratio.

Source: Mortality Information System and Live Births Systems.

**Figure 2.**
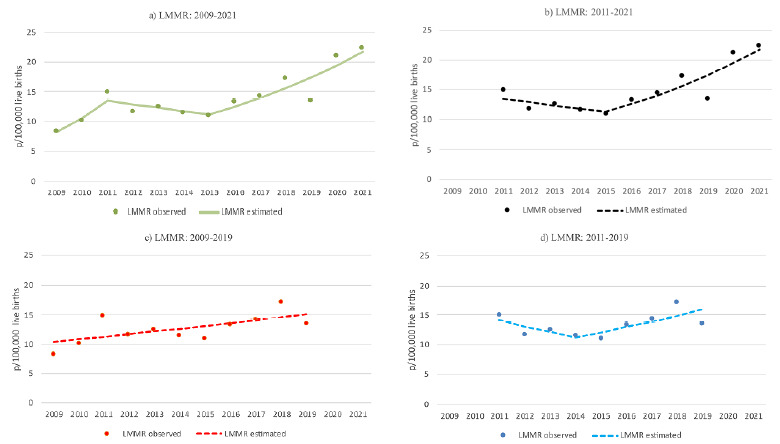
Trend of late maternal mortality ratio per 100,000 live births, Rio de Janeiro State, 2009 to 2021.

LMMR: late maternal mortality ratio.

Source: Mortality Information System and Live Births System.

## DISCUSSION

In the state of Rio de Janeiro, from 2009 to 2021, the MMR showed a downward trend until 2019; however, with the onset of the COVID-19 pandemic (2020-2021), the trend shifted sharply upward. The COVID-19 pandemic appears to have significantly influenced the temporal pattern of MMR. When the final two-year period is excluded, the trend reverts to a downward trajectory, even with the inclusion of the influenza A H1N1 pandemic years, and becomes stationary when both pandemic biennia are excluded. When only the initial two-year period is excluded, a declining trend is observed until the beginning of the COVID-19 pandemic, at which point the trend turns upward.

Mendonça et al.^5^ estimated an annual reduction of 1.3% in the MMR due to obstetric causes in the state of Rio de Janeiro between 2006 and 2018, and identified a peak in 2009, attributed to the H1N1 pandemic. This initial peak during the influenza pandemic years influenced the subsequent downward trend observed prior to the COVID-19 pandemic. Similarly, in Argentina, from 1980 to 2017, the downward trend in MMR was abruptly and temporarily interrupted in 2009 due to the H1N1 pandemic, after which the decline resumed at a faster pace^25^.

Excess maternal mortality during the COVID-19 pandemic years was reported in several studies, including a hospital cohort study of pregnant women in the state of Rio de Janeiro.^13^ The increase in maternal mortality was also observed in a national study using data from SIM^7^, and in a multinational cohort study involving pregnant women from 18 countries, where COVID-19 was identified as a risk factor for both maternal mortality and severe maternal morbidity (including Brazil)^26^. In the United States, the MMR increased by 18.4% from the year before to the first year of the COVID-19 pandemic, with the MMR rising from 20.1 per 100,000 live births in 2019 to 23.8 in 2020^27^.

Regarding the LMMR, the temporal behavior observed in the present study differed from that of the MMR. Over the entire period, the trend was predominantly upward, although it remained stationary between 2011 and 2015. Removing the H1N1 pandemic years at the beginning of the period changed the trend’s direction, from stationary to upward from 2015 onward, with the final biennium (COVID-19) maintaining an upward trajectory. When excluding the final biennium, the trend was downward until 2014, followed by an increase. This pattern of late maternal mortality suggests that the H1N1 pandemic had a greater influence than the COVID-19 pandemic. In the absence of the influenza A/H1N1 pandemic years, the junction points (2014 and 2015) align with the Zika virus epidemic (2014-2016), which, combined with the country’s political and socioeconomic crisis, contributed to the intensification of the decline in the frequency of LB^28^, the denominator of LMMR, thereby favoring an increase in this indicator. However, this was not observed in the evolution of the MMR, which shares the same denominator as the LMMR. Therefore, the increase in LMMR, regardless of the presence of the H1N1 and COVID-19 pandemics, may reflect a real increase in the risk of maternal death and/or improved reporting of maternal deaths. The epidemiological investigation of deaths among women of childbearing age and maternal deaths, regulated by the Ministry of Health in 2008 (Ordinance No. 1.119 of June 5, 2008), and conducted throughout the analyzed period, may have been a significant factor contributing to the rise in the frequency of late maternal deaths, as these are more likely to be underreported compared to deaths occurring within 42 days postpartum^17^. A similar trend was observed in the national analysis from 2010 to 2019^19^, although the magnitude of the annual percentage growth was lower: 3.8% in Rio de Janeiro compared to 9.8% in the country for the period from 2009 to 2019. Despite this, the LMMR in Rio de Janeiro was higher, reaching 13.4/100,000 LB in 2019, while in Brazil the figure was 5.6/100,000 LB. Between 2010 and 2019, both in Rio de Janeiro and the country as a whole, 2018 recorded the highest number of late maternal deaths^19^. Additionally, the negative correlation between the trends of MMR (decreasing) and LMMR (increasing), excluding the COVID-19 pandemic years, observed in our study, may reflect a shift in maternal deaths beyond 42 days postpartum due to increased survival, resulting from advances in life support technology, as seen in developed countries^15^ ^,^ ^20^. However, this trend was not observed when both pandemics were excluded.

The H1N1 pandemic coincided with the initiation of investigations into maternal deaths and deaths among women of childbearing age, at a time when the percentage of investigations was still low. In the country, the number of maternal deaths due to respiratory diseases quadrupled in 2009 compared to the average of previous years, and this increase was attributed to the influenza A/H1N1 pandemic^29^.

The excess maternal mortality observed in 2021, compared to the previous year, can be attributed to the high transmissibility of the SARS-CoV-2 Gamma variant, which was predominant during the second wave of COVID-19, as well as the delay in prioritizing pregnant and postpartum women for vaccination^7^ ^,^ ^30^. In five locations in Bogotá, Bolivia, 10 and 17% of maternal deaths in 2020 (total=10) and 2021 (total=23), respectively, were attributed to COVID-19^31^. Although the number of maternal deaths was higher in 2021 than in 2020, the proportions in Bolivia remained lower than those recorded in the state of Rio de Janeiro. At the national level, 18% of maternal deaths in 2020 and 43% in 2021 were associated with COVID-19^12^.

Within the study period, the year 2018 stands out due to a decrease in the MMR and an increase in the LMMR. This rise in LMMR in 2018 was also observed at the national level^19^. This finding suggests the possibility of misclassification related to the timing of death recorded on the DC, underscoring the importance of accurately completing the fields pertaining to the timing of death and the underlying cause.

Studies on late maternal mortality are essential to assess the magnitude of the problem and guide responses through appropriate health policies^17^ ^,^ ^18^ ^,^ ^19^. In the present study, based on data from SIM, the proportion of late maternal deaths (ICD-10 code O96) in the state of Rio de Janeiro increased from 7.4% in 2009 to 12.7% in 2021. Prior to the pandemics, a cross-sectional study conducted in Brazilian capitals had already identified 13.8% of late maternal deaths among confirmed maternal deaths in 2002^32^. In Recife (Pernambuco - PE), among 171 maternal deaths that occurred between 2006 and 2017, 20.5% were classified as late^18^.

In some publications, late maternal mortality is defined as the sum of deaths coded as O96 and O97 (obstetric sequelae occurring more than one year after delivery), which complicates the comparison of results, even though the frequency of deaths from obstetric sequelae is low and lower than that of late maternal deaths. In an ecological study conducted in seven countries in the Americas between 1999 and 2013, the highest proportion of late maternal deaths and deaths due to obstetric sequelae among total maternal deaths was observed in the United States (18.7%), followed by Cuba (11.5%), Argentina (approximately 6%), Brazil and Canada (approximately 5%), Mexico (3.6%), and Colombia (2.4%)^33^. The trend in late maternal mortality and deaths from obstetric sequelae in the Americas was upward: global mean APC (estimated using Poisson regression models) of 12.7%, and 9.7% for Brazil^33^.

In 2018, of the 935 maternal deaths recorded in the United States, 29.6% were classified as late maternal deaths (code O96), and the MMR and LMMR were 17.4 and 7.2 per 100,000 LB, respectively^16^. In comparison, in the same year, 21.8% of maternal deaths in the state of Rio de Janeiro were late, and the MMR and LMMR were 61.5 and 17.2 per 100,000 LB, respectively, corresponding to 3.5 and 2.4 times the values observed in the United States.

Pandemics contribute to the deterioration of healthcare systems and to excess mortality, even when infectious diseases are not explicitly listed among the causes of death^6^ ^,^ ^7^. Considering the national landscape of maternal mortality, it appears unlikely that the Sustainable Development Goal target of reducing the maternal mortality ratio to 30 per 100,000 live births by 2030 will be achieved^34^.

Surveillance and statistical reporting of late maternal deaths are complex processes^35^, and the challenge of minimizing or eliminating underreporting and misclassification remains^2^. Nonetheless, some progress has been made. In 2022, 54% of the 120 countries and territories included in the World Health Organization (WHO) maternal mortality statistics reported on late maternal mortality (ICD-10)^2^.

Classification errors of maternal and late maternal deaths may have occurred in this study due to limitations in the accuracy of certifying the underlying cause of death and the completion of field 37 (time of death in the pregnancy-puerperal cycle) on the DC. Additionally, changes in mortality trends estimated by joinpoint regression are constrained by the number of observations in the historical series, potentially limiting the robustness of analyses when pandemic biennia are excluded.

A notable strength of this study is the analysis of maternal mortality trends both including and excluding pandemic years. While Vega et al.^14^ recommend excluding epidemic years from trend analyses of maternal mortality, the methodological approach adopted in this study - joinpoint regression modeling - does not support such exclusion. On the contrary, this method allowed for the observation of how trends varied with the inclusion of pandemic biennia, whether jointly or separately. Thus, the dual analysis approach contributed to a more comprehensive understanding of the temporal behavior of maternal mortality. Furthermore, the high coverage of the SIM and Sinasc^36^, along with the increased proportion of investigations into deaths of women of childbearing age and maternal deaths, from 80.9% in 2010 to 93.7% in 2021 in the state^37^, enhanced the reliability of the data and the accuracy of estimates of maternal and late maternal mortality risks.

Late maternal deaths are not included in the calculation of MMR, the primary indicator in maternal mortality statistics; however, the increasing relevance of this component in the overall risk of maternal death must be considered. The WHO recommends calculating the late MMR separately, with the possibility of aggregating it with the MMR up to 42 days postpartum, provided that the composition of the indicator is clearly stated^25^. In the 11^th^ revision of the ICD, maternal and late maternal deaths were grouped under the category of “comprehensive maternal deaths,” for which the corresponding indicator is the comprehensive or expanded MMR^38^. The systematic monitoring of this broader indicator should be incorporated into maternal mortality statistics. Additionally, follow-up during the late postpartum period is a key strategy for reducing preventable late maternal deaths^19^.
