# Supplementary material for: Maternal and late mortality trends, emphasizing the H1N1 and COVID-19 pandemics, in the state of Rio de Janeiro, Brazil, from 2009 to 2021
Source: Rev Bras Epidemiol. 2025 Jul 21;28:e250037. doi: 10.1590/1980-549720250037 (PMC12278847; doi:10.1590/1980-549720250037)
Supplement: Translation

# Translation: Tendência da mortalidade materna e materna tardia, com ênfase nas pandemias de H1N1 e COVID-19, estado do Rio de Janeiro, Brasil, de 2009 a 2021

**DOI:** 10.1590/1980-549720250037.2

**Authors:** Ana Lucia de Melo Bellizzi, Angela Maria Cascão, Alexandre dos Santos Brito, Sandra Costa Fonseca, Pauline Lorena Kale

**Affiliations:** I Universidade Federal do Rio de Janeiro, Instituto de Estudos em Saúde Coletiva, Programa de Pós-Graduação - Rio de Janeiro (RJ), Brasil.; II Secretaria Estadual de Saúde do Rio de Janeiro, Subsecretaria de Vigilância em Saúde - Rio de Janeiro (RJ), Brasil.; III Universidade Federal do Rio de Janeiro, Instituto de Estudos em Saúde Coletiva - Rio de Janeiro (RJ), Brasil.; IV Universidade Federal Fluminense, Instituto de Saúde Coletiva - Niterói (RJ), Brasil.

**Keywords:** Estudos de séries temporais, Mortalidade materna, Vírus da influenza A subtipo H1N1, COVID-19

## Abstract

**Objetivo:**

Estimar as tendências das razões de mortalidade materna (RMM) e mortalidade materna tardia (RMMT), em períodos com e sem as pandemias de H1N1 e COVID-19, no estado do Rio de Janeiro, Brasil, de 2009 a 2021.

**Métodos:**

Estudo ecológico de tendência temporal. Dados obtidos dos Sistemas de Informações sobre Mortalidade e Nascidos Vivos. Foram calculadas as RMM e RMMT anuais por 100 mil nascidos vivos (NV) e foi aplicado o modelo de regressão *joinpoint* para estimar a tendência.

**Resultados:**

Em 2009, a RMM foi 103,1, alcançando, em 2021, 152,4/100.000 NV, com redução anual de 3,3% (intervalo de confiança de 95% — IC95% -5,5; -1,7) até 2019, e ascensão de 51,2% (IC95% 23,5; 64,5) em 2020/21. Excluindo-se os anos da pandemia de COVID-19, observou-se declínio anual de 3,3% e, com a concomitante exclusão dos anos da pandemia de H1N1, estabilidade. As RMMT foram 8,3 (2009) e 22,2 (2021) p/100.000 NV, com crescimento anual de 28,2% (IC95% 11,8; 47,8) até 2011, mantendo-se estacionária de 2011 a 2015, seguida de ascensão de 11,7% até 2021; com a exclusão do biênio final, a tendência é ascendente (3,8%) e, com a exclusão também do biênio inicial, a tendência passou a ser descendente (7%) até 2014 e a seguir ascendente (8,2%).

**Conclusão:**

Houve mudança de tendência com a incorporação separada ou conjunta dos biênios pandêmicos: sem as pandemias, a mortalidade materna seria estacionária, apesar das ações de prevenção do óbito materno, e a materna tardia seria descendente até 2014 e depois ascendente, creditando-se tais tendências em parte à melhoria da investigação de óbito.

## INTRODUÇÃO

Mundialmente, a cada dois minutos uma mulher morre em razão de parto ou complicações da gravidez^1^, correspondendo à razão de mortalidade materna (RMM - risco de morte materna da gestação até 42 dias após o parto) de 223 por 100 mil nascidos vivos (NV) em 2020^2^. Nesse mesmo ano, no Brasil, a RMM foi 72 por 100 mil NV, cerca de 16 vezes superior à da Alemanha e oito vezes à de Cuba, indicando o potencial de redução^3^. De 2000 a 2020, a redução anual da RMM global foi, em média, 2,1%, enquanto na América Latina e Caribe a tendência foi de estagnação^3^.

As pandemias de influenza (H1N1) em 2009 e 2010 e de COVID-19 (SARS-CoV-2), cerca de dez anos depois (no Brasil, a partir de 2020), impactaram a mortalidade materna^4^ ^,^ ^5^ ^,^ ^6^, embora não expliquem totalmente as tendências observadas^3^. Em decorrência das alterações anatômicas e fisiológicas nos sistemas cardiovascular, respiratório, imunológico e da coagulação na gravidez, a mulher torna-se mais susceptível às pneumonias virais, principalmente em momentos de pandemia, que agravam os problemas de assistência já existentes^6^ ^,^ ^7^.

A declaração de transmissão sustentada do vírus influenza A (H1N1), nacionalmente, ocorreu em 19 de julho de 2009. Em 2009, o país registrou quase 60 mil casos da doença e 2.146 mortes, e em 2010, com a vacinação, o número de mortes caiu para cerca de cem^8^ ^,^ ^9^. Mais de 10% do total de mortes provocadas pela pandemia de gripe A/H1N1 ocorreu entre gestantes, ainda que a maioria delas fosse saudável^10^. No primeiro trimestre de 2010 a vacina monovalente anti-H1N1 foi distribuída em todo o país^9^. Dez anos depois, em junho de 2020, o país contava com 124 mortes de mulheres no ciclo gravídico puerperal devidas à COVID-19, correspondendo a 12,7% de mortes entre as gestantes internadas por síndrome respiratória aguda grave - SRAG^11^. Dos óbitos maternos nacionais, 18 e 43% em 2020 e 2021, respectivamente, estiveram associados à COVID-19^12^. A imunização antiCOVID-19 completa em gestantes foi altamente protetora de morbidade materna grave e mortalidade materna na coorte de gestantes assistidas em hospital geral público no estado do Rio de Janeiro^13^. Em ambas as pandemias virais, mulheres no ciclo gravídico-puerperal foram consideradas grupo de alto risco para morbidade materna grave e mortalidade materna^11^ ^,^ ^12^ ^,^ ^13^. Para Vega et al.^14^, situações de pandemia não deveriam ser levadas em consideração na análise da série histórica do comportamento da mortalidade materna, pois são específicas, assim como as grandes catástrofes, agindo como um fator externo que independe da gestão pública e acometendo populações despreparadas para o manuseio do problema^14^.

Com a efetividade da tecnologia aprimorada de suporte à vida, o limite de 42 dias após o parto para a mortalidade materna tornou-se sem sentido^15^. Nos EUA, a introdução da informação indicando gravidez no último ano antes da morte na Declaração de Óbito (DO) levou a um aumento da detecção da mortalidade materna tardia (de 43 dias até um ano após o parto)^16^.

No Brasil, além da incorporação dessa informação na DO, a investigação epidemiológica dos óbitos de mulheres em idade fértil e maternos, desde 2008, tem sido importante para minimizar a subenumeração de mortes maternas e maternas tardias no Brasil^17^ ^,^ ^18^. De 2010 a 2019, a tendência da mortalidade materna tardia no país foi ascendente, de 2,2 a 5,6/100.000 NV, com velocidade de 9,8% ao ano e disparidades regionais^19^. Na cidade de São Paulo (SP) e no estado do Paraná, as proporções de mortes tardias entre os óbitos maternos foram, respectivamente, 13,4 e 12,1%^17^. Há grande potencial de prevenção desses óbitos, dada a existência de tratamentos efetivos em tempo hábil, apontando a necessidade de estender o tempo de acompanhamento das mulheres no puerpério, principalmente as que apresentam maior risco^17^ ^,^ ^18^ ^,^ ^19^. Negligenciar óbitos maternos tardios leva à fragmentação dos cuidados e à perda de oportunidades de prevenção da morte^20^.

O objetivo deste estudo foi analisar as tendências da RMM e de mortalidade materna tardia (RMMT) em períodos com e sem as pandemias de influenza A (H1N1) e de COVID-19, no estado do Rio de Janeiro, de 2009 a 2021.

## MÉTODOS

Foi desenvolvido um estudo ecológico de tendência temporal da RMM e da RMMT anuais no estado do Rio de Janeiro, de 2009 a 2021.

Mortes maternas referem-se àquelas ocorridas durante a gestação, o parto e até 42 dias após o término da gestação, independentemente da duração ou da localização da gravidez, devidas a qualquer causa relacionada com ou agravada pela gravidez ou por medidas relacionadas a ela, porém não devidas a causas acidentais ou incidentais^21^. São incluídos como causas de morte materna os códigos O00.0 a O08.9, O11 a O23.9, O24.4, O26.0 a O92.7, D39.2, E23.0, F53 e M83.0 (causas obstétricas diretas), O10.0 a O10.9, O24.0 a O24.3, O24.9, O25, O98.0 a O99.8, A34, B20 a B24 (causas obstétricas indiretas) e O95 (causas não especificadas) do capítulo XV da CID-10, e adicionalmente, os códigos A34, B20 a B24, D39.2, E23.0, F53 e M83.0 fora do capítulo XV, quando essas causas tiverem levado a óbito durante o ciclo gravídico-puerperal^21^.

Mortes maternas tardias são mortes por qualquer causa obstétrica que ocorrem em mais de 42 dias, mas em menos de um ano após o parto, correspondendo ao código O96 da Classificação Estatística Internacional de Doenças e Problemas Relacionados à Saúde da Décima Revisão - CID-10^21^.

As fontes de dados secundários foram os Sistemas de Informações sobre Mortalidade (SIM) e sobre Nascidos Vivos (Sinasc) da Secretaria Estadual de Saúde do Rio de Janeiro.

As distribuições absoluta e relativa percentuais dos óbitos maternos e maternos tardios foram descritas anualmente. Foram calculadas as RMM e RMMT anuais por 100 mil NV (quociente entre óbitos maternos e NV, multiplicado por 100.000).

A opção de iniciar a análise temporal em 2009 deveu-se à dispensa do uso de fator de correção calculado apenas para RMM, no período anterior, no estado do Rio de Janeiro^4^. As séries temporais de mortalidade materna foram avaliadas para o período completo (2009 a 2021), sem o biênio inicial da pandemia de influenza A/H1N1 (2011 a 2021), sem o biênio final da pandemia de COVID-19 (2009 a 2019) e sem ambos os biênios pandêmicos (2011 a 2019).

Para estimar a tendência, utilizou-se o modelo de regressão *joinpoint*, que identifica mudanças significativas da tendência (pontos de inflexão) e ajusta, em escala logarítmica, tendências lineares^22^ ^,^ ^23^ ^,^ ^24^. A seleção de modelos (considerando-se o número de pontos de junção) foi feita por meio do critério de informação bayesiano ponderado (W-BIC). Para representar a direção e magnitude da tendência, foi estimada a variação percentual anual - VPA (nível de significância estatística de 0,05). As análises foram realizadas na ausência de autocorrelação e considerando-se autocorrelação serial de primeira ordem. Foi selecionado o modelo que melhor descreveu a estrutura de tendência da série histórica.

Este estudo integra a pesquisa aprovada pelo Comitê de Ética em Pesquisa da Universidade Federal Fluminense (Certificado de Apresentação para Apreciação Ética - CAAE n^o^ 713230230000005243; parecer 6.592.725, de 19 de dezembro de 2023).

### Declaração de disponibilidade de dados

Todo o conjunto de dados que dá suporte aos resultados deste estudo foi publicado no próprio artigo.

## RESULTADOS

Em 2009 o número de NV no estado do Rio de Janeiro foi 217.280 e, em 2021, 188.989, correspondendo a uma redução de 13% (Tabela 1). Destaca-se, entretanto, que até 2015 ocorreu ligeiro aumento (8,5%) da frequência de NV e, a partir de então, declínio de 25,7% (Tabela 1).

**Tabela 1.** Distribuição absoluta e percentual dos óbitos maternos e maternos tardios, n^o^ de nascidos vivos, razão de mortalidade materna* e razão de mortalidade materna tardia^†^, estado do Rio de Janeiro, 2000 a 2021.

Ano	N^o^ nascidos vivos	N^o^ óbitos maternos (totais)	Mortalidade materna*			Mortalidade materna tardia^†^		
			N^o^ óbitos	% óbitos	RMM	N^o^ óbitos	% óbitos	RMMT
2009	217.280	242	224	92,6	103,1	18	7,4	8,3
2010	215.916	211	189	89,6	87,5	22	10,4	10,2
2011	221.277	197	164	83,2	74,1	33	16,8	14,9
2012	223.422	212	186	87,7	83,3	26	12,3	11,6
2013	224.745	221	193	87,3	85,9	28	12,7	12,5
2014	234.281	202	175	86,6	74,7	27	13,4	11,5
2015	237.591	193	167	86,5	70,3	26	13,5	10,9
2016	219.711	185	156	84,3	71,0	29	15,7	13,2
2017	223.415	205	173	84,4	77,4	32	15,6	14,3
2018	221.045	174	136	78,2	61,5	38	21,8	17,2
2019	208.677	177	149	84,2	71,4	28	15,8	13,4
2020	199.550	229	187	81,7	93,7	42	18,3	21
2021	188.989	330	288	87,3	152,4	42	12,7	22,2

RMM: Razão de mortalidade materna por 100 mil nascidos vivos e razão de mortalidade materna tardia por 100 mil nascidos vivos.

*Causas de morte materna do Capítulo XV e fora do Capítulo XV - CID-10 até 42 dias após o parto; 10: O96); ^†^Morte Materna Tardia - de 43 dias até menos de um ano após o parto (CID-10: O96).

Fonte: Sistema de Informações sobre Mortalidade e sobre Nascidos Vivos.

Ocorreram 242 e 330 óbitos maternos totais (incluindo óbitos maternos tardios), respectivamente, no início e no final do período, sendo a menor frequência 174 óbitos, em 2018, e a maior em 2021 (Tabela 1). Predominam óbitos maternos até 42 dias após o parto, observando-se considerável aumento da proporção dos óbitos maternos tardios (43 dias até um ano após o parto), de 7,4% em 2009 para 18,3% em 2020. Em 2018, observou-se o maior valor da RMMT antes da pandemia de COVID-19. A RMM apresentou os valores mais elevados no biênio inicial e final do período, variando de 61,5 (2018) a 152,4 (2021) por 100 mil NV (Tabela 1)t.

As tendências estimadas, pontos de junção (mudança da tendência) e o coeficiente de VPA das RMM e RMMT, com ou sem os anos pandêmicos, são apresentados de forma sumária na Tabela 2. Essas medidas foram obtidas considerando-se autocorrelação de primeira ordem, pois essa abordagem descreveu melhor a tendência da série histórica.

**Tabela 2.** Tendência da razão de mortalidade materna e razão de mortalidade materna tardia por 100 mil nascidos vivos, estado do Rio de Janeiro, 2009 a 2021.

Período	Ponto de junção	Segmentos	Razão de mortalidade		Variação percentual anual (IC95%)*	Tendência
			Início	Final		
Materna^†^						
2009-2021	2019	2009-2019	103,1	71,4	-3,3 (-5,5; -1,7)	descendente
		2019-2021	71,4	152,4	51,2 (23,5; 64,5)	ascendente
2011-2021	2019	2011-2019	74,1	71,4	-2,7 (-5,7; 0,5)	descendente
		2019-2021	71,4	152,4	49,0 (23,5; 62,6)	ascendente
2009-2019	-	-	103,1	71,4	- 3,3 (-5,4; -1,3)	descendente
2011-2019	-	-	74,1	71,4	- 2,6 (-5,5; 0,2)	estacionária
Tardia^‡^						
2009-2021	2009	2009-2011	8,3	14,9	28,3 (11,8; 47,8)	ascendente
	2011	2011-2015	14,9	10,9	-4,6 (-11,7; 0,4)	estacionária
	2015	2015-2021	10,9	22,2	11,7 (8,7; 17,6)	ascendente
2011-2021	2015	2011-2015	14,9	10,9	- 4,4 (-17,9; 2,2)	estacionária
		2015-2021	10,9	22,2	11,7 (7,9; 20,4)	ascendente
2009-2019	-	-	8,3	13,4	3,8 (0,2; 8,1)	ascendente
2011-2019	2014	2011-2014	14,9	11,5	- 7,0 (-16,8; -0,9)	descendente
		2014-2019	11,5	13,4	8,2 (4,7; 15,4)	ascendente

*IC95%: Intervalo de confiança de 95%; ^†^Causas de morte materna do Capítulo XV e fora do Capítulo XV - CID-10 até 42 dias após o parto; 10: O96); ^‡^Morte materna tardia - de 43 dias até menos de um ano após o parto (CID-10: O96).

Fonte: Sistema de Informações sobre Mortalidade e sobre Nascidos Vivos.

No período completo de 2009 a 2021, observamos tendência segmentada da RMM, incialmente com declínio anual de 3,3% até 2019 (ponto de junção), seguida de aumento anual de 51,2% nos dois anos finais da série (pandemia de COVID-19 - Tabela 2). Sem o biênio inicial, a tendência foi descendente até 2019 (VPA=-2,7%) e ascendente a partir de então (VPA=49%). Sem o biênio final, observou-se declínio anual de 3,3% e, sem os dois biênios pandêmicos (inicial e final), observou-se estabilidade (Tabela 2).

Para RMMT, considerando-se o período completo, ocorreram três mudanças de tendência: aumento de 28,3% ao ano até 2011, seguido de um período estacionário e, a partir de 2015, aumento anual de 11,7% (Tabela 2). Com a exclusão do biênio inicial, a tendência foi estacionária até 2015 e ascendente a partir de então (VPA=11,7%). Sem o biênio final, não houve ponto de junção e a tendência foi ascendente (VPA=3,8%). Retirando os dois biênios extremos, o declínio foi de 7% ao ano até 2014, seguido de aumento anual de 8,2% até 2019 (Tabela 2).

A representação gráfica das tendências observadas e estimadas da mortalidade materna e tardia de 2009 a 2021 e aquelas referentes às simulações na ausência das pandemias são apresentadas nas Figuras 1 (RMM) e 2 (RMMT).

**Figura 1.** Tendência da razão de mortalidade materna por 100 mil nascidos vivos, estado do Rio de Janeiro, 2009 a 2021.

RMM: razão de mortalidade materna.

Fonte: Sistema de Informações sobre Mortalidade e sobre Nascidos Vivos.

**Figura 2.** Tendência da razão de mortalidade materna tardia por 100 mil nascidos vivos, estado do Rio de Janeiro, 2009 a 2021.

RMMT: razão de mortalidade materna tardia.

Fonte: Sistema de Informações sobre Mortalidade e sobre Nascidos Vivos.

## DISCUSSÃO

No estado do Rio de Janeiro, no período de 2009 a 2021, sem exclusão dos anos pandêmicos na análise, a tendência da RMM foi descendente até 2019; com a pandemia de COVID-19 (2020/2021), passou a ser acentuadamente ascendente. Para a RMM, a pandemia de COVID-19 parece influenciar sobremaneira o comportamento temporal. Quando se exclui o biênio final (pandemia de COVID-19), a tendência passa a ser descendente considerando-se a manutenção dos anos pandêmicos de influenza A H1N1, e estacionária ao excluí-los. Quando se exclui apenas o biênio inicial, observa-se declínio até a pandemia de COVID-19, quando se inicia a tendência ascendente.

Mendonça et al.^5^ estimaram a tendência de redução de 1,3% ao ano da RMM por causas obstétricas do estado do Rio de Janeiro de 2006 a 2018, e observaram um pico do indicador em 2009, atribuído à pandemia de H1N1. Esse pico inicial nos anos de pandemia de influenza influencia o comportamento de declínio subsequente, que antecede a pandemia de COVID-19. Na Argentina, de 1980 a 2017, a tendência de declínio da RMM foi interrompida abrupta e transitoriamente em 2009, diante da pandemia de H1N1, voltando a decrescer de forma mais acelerada a partir de então^25^.

O excesso de mortalidade materna nos anos de pandemia de COVID-19 foi relatado, por exemplo, no estudo de coorte hospitalar de gestantes no estado do Rio de Janeiro^13^. A elevação do indicador foi observada no estudo nacional com dados do SIM^7^ e, na coorte multinacional de gestantes 18 países, a COVID-19 foi considerada fator de risco tanto para mortalidade quanto morbidade materna grave (incluindo o Brasil)^26^. Nos EUA, a ascensão da RMM entre o ano precedente e o primeiro ano da COVID-19 foi de 18,4% (RMM por 100.000 NV: 20,1 em 2019 e 23,8 em 2020)^27^.

Quanto à RMMT, o comportamento temporal observado no presente estudo foi distinto da RMM. Considerando-se o período completo, a tendência foi predominantemente ascendente, apesar de estacionária entre 2011 e 2015. A retirada dos anos de pandemia de H1N1, no início do período, mudou a direção da tendência, de estacionária para ascendente a partir de 2015, mantendo-se ascendente no biênio final (COVID-19), e descendente até 2014 seguida de ascensão, quando o biênio final é excluído. Esse comportamento temporal da mortalidade materna tardia sugere maior influência da pandemia de H1N1 quando comparada à pandemia de COVID-19. Com a ausência dos anos pandêmicos de influenza A/H1N1, os pontos de junção (2014 e 2015) coincidem com a epidemia de zika vírus (2014/2016), que associada à crise política e socioeconômica do país contribuiu para a intensificação do declínio da frequência de NV^28^, denominador da RMMT, favorecendo assim o aumento desse indicador. Esse comportamento, entretanto, não foi observado na evolução da RMM, cujo denominador é o mesmo da RMMT. Nesse sentido, o aumento da RMMT, independentemente da presença das pandemias de H1N1 e de COVID-19, pode estar refletindo um aumento real de risco de morte e/ou o conhecimento oficial do número de mortes maternas. A investigação epidemiológica dos óbitos de mulheres em idade fértil e maternos regulamentada pelo Ministério da Saúde em 2008 (portaria n^o^ 1.119, de 05 de junho de 2008) e, portanto, realizada em todo o período analisado pode ter sido a maior contribuição para a elevação da frequência de óbitos maternos tardios, cuja subnotificação é maior do que as ocorridas até 42 dias do puerpério^17^. O mesmo comportamento foi observado na análise nacional de 2010 a 2019^19^, porém a magnitude do crescimento percentual anual foi inferior: 3,8% no Rio de Janeiro, comparada a 9,8% no país, considerando-se o período de 2009 a 2019. Mesmo assim, as RMMT do Rio de Janeiro foram superiores, atingindo 13,4 em 2019, enquanto no Brasil o valor foi 5,6/100.000 NV. Entre 2010 e 2019, tanto no Rio de Janeiro como no país, 2018 foi o ano com maior registro de óbitos maternos tardios^19^. Adicionalmente, a relação negativa entre as tendências da RMM (decrescente) e da RMMT (ascendente), excluídos os anos pandêmicos de COVID-19, observada nos nossos resultados, poderia estar refletindo o deslocamento dos óbitos maternos para além de 42 dias após o parto em razão da ampliação da sobrevida, resultante de melhorias da efetividade da tecnologia de suporte à vida, como nos países desenvolvidos^15^ ^,^ ^20^. Entretanto, esse comportamento não foi observado na ausência de ambas as pandemias.

A pandemia de H1N1 coincidiu com o início das investigações de óbitos maternos e de mulheres em idade fértil, quando o percentual de investigações ainda era baixo. No país, o número de mortes maternas por doenças do aparelho respiratório quadriplicou em 2009 em comparação à média dos anos anteriores, sendo atribuído à pandemia de influenza A/H1N1^29^.

O excesso de mortalidade materna em 2021, comparado ao ano anterior, pode ser explicado pelo alto poder de disseminação da variante Gama do SARS-CoV-2, predominante na segunda onda da COVID-19, e pelo atraso na inclusão das gestantes e puérperas como grupo prioritário para vacinação^7^ ^,^ ^30^. Em cinco localidades da cidade de Bogotá, Bolívia, 10 e 17% das mortes maternas de 2020 (total=10) e 2021 (total=23), respectivamente, foram atribuídas à COVID-19^31^. Embora a magnitude em 2021 também seja superior à de 2020, as proporções foram inferiores às do estado do Rio de Janeiro. Em 2020 e 2021, 18% (2020) e 43% (2021) dos óbitos maternos nacionais estiveram associados à COVID-19^12^.

No período estudado, chama atenção o ano de 2018, com queda da RMM e aumento da RMMT. Esse aumento da RMMT em 2018 também foi observado no país^19^. Esse achado sugere a presença de erro no registro do campo referente ao momento de ocorrência do óbito na DO, ressaltando a importância do correto preenchimento com relação ao momento da morte e à causa básica.

Estudos sobre mortalidade materna tardia podem dimensionar a magnitude do problema e auxiliar o seu enfrentamento com base em políticas de saúde^17^ ^,^ ^18^ ^,^ ^19^. No presente estudo, com base nos dados do SIM, a proporção de mortes maternas tardias (código O96 da CID-10) aumentou de 2009 (7,4%) a 2021 (12,7%) no estado do Rio de Janeiro. Anteriormente às pandemias, um estudo transversal nas capitais brasileiras já havia quantificado 13,8% de mortes maternas tardias entre os óbitos maternos confirmados em 2002^32^. Em Recife (PE), dos 171 óbitos por causas maternas ocorridos entre 2006 e 2017, 20,5% eram tardios^18^.

Em algumas publicações, a mortalidade materna tardia foi definida como a soma de causas de morte referentes aos códigos O96 e O97 (sequelas obstétricas ocorrida após um ano do parto), o que dificulta a comparação dos resultados, mesmo considerando-se que a frequência dos óbitos por sequelas obstétricas é baixa e inferior à frequência de mortes tardias. No estudo ecológico em sete países das Américas de 1999 a 2013, a maior proporção de mortes tardias e sequelas obstétricas em meio ao total de óbitos maternos foi para os EUA (18,7%), seguidos de Cuba (11,5%), Argentina (cerca de 6%), Brasil e Canadá (cerca de 5%), México (3,6%) e Colômbia (2,4%)^33^. A tendência da mortalidade materna tardia e sequelas obstétricas nas Américas foi crescente: a VAP média (estimada por modelos de regressão de Poisson) global foi de 12,7% e, para o Brasil, de 9,7%^33^.

Em 2018, dos 935 óbitos maternos nos EUA, 29,6% eram tardios (O96) e as RMM e RMMT foram respectivamente 17,4 e 7,2 por 100.000 NV^16^. Quando comparados aos resultados do presente estudo nesse mesmo ano, 21,8% dos óbitos maternos do estado do Rio de Janeiro eram tardios, e as razões de mortalidade materna foram 61,5 (RMM) e 17,2 (RMMT) por 100.000 NV, correspondendo a 3,5 e 2,4 vezes os valores das razões de mortalidade materna estadunidenses.

Pandemias contribuem para o agravamento dos problemas de assistência e o excesso de mortes, mesmo sem menção da doença infecciosa entre as causas de morte^6^ ^,^ ^7^. Diante do panorama nacional de mortalidade materna, dificilmente será cumprida a meta acordada do Objetivo do Desenvolvimento Sustentável de redução da mortalidade materna para 30 por 100 mil nascidos vivos em 2030^34^.

A vigilância e as estatísticas de morte materna tardia não são triviais^35^, e o desafio de minimizar ou erradicar o sub-registro e o erro de classificação da morte materna tardia permanece^2^. Entretanto, observam-se algumas melhorias. Em 2022, 54% dos 120 países e territórios incluídos nas estatísticas de mortalidade materna da Organização Mundial da Saúde (OMS) analisaram a mortalidade materna tardia (CID-10)^2^.

Falhas de classificação do óbito materno e materno tardio podem ter ocorrido no estudo em função da qualidade da certificação da causa básica e do preenchimento do campo 37 (momento da ocorrência do óbito no ciclo gravídico puerperal) da DO. Mudanças de tendência da mortalidade estimadas pela regressão *joinpoint* são limitadas ao número de observações da série histórica, o que pode ter penalizado as análises com a exclusão dos biênios pandêmicos.

Como ponto forte do estudo, destacamos a análise de tendências com e sem os anos pandêmicos. Vega et al.^14^ recomendam não considerar anos epidêmicos na análise de tendência da mortalidade materna. Metodologicamente, a modelagem de regressão *joinpoint* aplicada aos dados do presente estudo não recomenda a exclusão de anos epidêmicos, pois observamos o quanto as tendências se modificaram com a incorporação dos biênios pandêmicos, em conjunto ou separadamente. Acredita-se que analisar com e sem o período pandêmico, como realizado no presente estudo, contribuiu para melhor compreensão do comportamento temporal da mortalidade materna. A boa cobertura do SIM e Sinasc^36^, além do aumento da proporção de investigações de óbitos de mulheres em idade fértil e maternos, passando de 80,9% em 2010 para 93,7% em 2021 no estado^37^, contribuiu para um conhecimento mais fidedigno do risco de mortalidade materna e materna tardia.

As mortes maternas tardias não são contabilizadas para o cálculo da RMM, principal indicador das estatísticas de mortalidade materna, porém há que considerar a importância crescente desse componente no risco de morte. A OMS recomenda o cálculo da RMM tardia separadamente, podendo ser agregada à RMM até 42 dias, se esclarecida a composição do indicador^25^. Na 11^a^ revisão da CID, óbitos maternos e óbitos maternos tardios foram combinados em um grupo definido como “*comprehensive maternal deaths*”, cujo respectivo indicador corresponderia à RMM abrangente ou ampliada^38^. O seu monitoramento deve ser sistematicamente realizado nas estatísticas de mortalidade materna. O acompanhamento das mulheres no puerpério tardio é uma estratégia importante para a redução de mortes tardias por causas evitáveis^19^.
